# Leopard syndrome

**DOI:** 10.1186/1750-1172-3-13

**Published:** 2008-05-27

**Authors:** Anna Sarkozy, Maria Cristina Digilio, Bruno Dallapiccola

**Affiliations:** 1 IRCCS-CSS, San Giovanni Rotondo and CSS-Mendel Institute, Viale Regina Elena 261, 00198, Rome, Italy; 2 Medical Genetics, Bambino Gesù Hospital, Piazza San't Onofrio 4, 00165 Rome, Italy; 3 Department of Experimental Medicine and Pathology, University "La Sapienza", Viale Regina Elena 261, 00198, Rome, Italy

## Abstract

LEOPARD syndrome (LS, OMIM 151100) is a rare multiple congenital anomalies condition, mainly characterized by skin, facial and cardiac anomalies. LEOPARD is an acronym for the major features of this disorder, including multiple *L*entigines, *E*CG conduction abnormalities, *O*cular hypertelorism, *P*ulmonic stenosis, *A*bnormal genitalia, *R*etardation of growth, and sensorineural *D*eafness. About 200 patients have been reported worldwide but the real incidence of LS has not been assessed. Facial dysmorphism includes ocular hypertelorism, palpebral ptosis and low-set ears. Stature is usually below the 25^th ^centile. Cardiac defects, in particular hypertrophic cardiomyopathy mostly involving the left ventricle, and ECG anomalies are common. The lentigines may be congenital, although more frequently manifest by the age of 4–5 years and increase throughout puberty. Additional common features are *café-au-lait spots *(CLS), chest anomalies, cryptorchidism, delayed puberty, hypotonia, mild developmental delay, sensorineural deafness and learning difficulties. In about 85% of the cases, a heterozygous missense mutation is detected in exons 7, 12 or 13 of the *PTPN11 *gene. Recently, missense mutations in the *RAF1 *gene have been found in two out of six *PTPN11*-negative LS patients. Mutation analysis can be carried out on blood, chorionic villi and amniotic fluid samples. LS is largely overlapping Noonan syndrome and, during childhood, Neurofibromatosis type 1-Noonan syndrome. Diagnostic clues of LS are multiple lentigines and CLS, hypertrophic cardiomyopathy and deafness. Mutation-based differential diagnosis in patients with borderline clinical manifestations is warranted. LS is an autosomal dominant condition, with full penetrance and variable expressivity. If one parent is affected, a 50% recurrence risk is appropriate. LS should be suspected in foetuses with severe cardiac hypertrophy and prenatal DNA test may be performed. Clinical management should address growth and motor development and congenital anomalies, in particular cardiac defects that should be monitored annually. Hypertrophic cardiomyopathy needs careful risk assessment and prophylaxis against sudden death in patients at risk. Hearing should be evaluated annually until adulthood. With the only exception of ventricular hypertrophy, adults with LS do not require special medical care and long-term prognosis is favourable.

## Disease name and synonyms

Leopard syndrome (LS, OMIM 151100) is named based on an acronym, mnemonic for the major features of this disorder: multiple *L*entigines, *E*CG conduction abnormalities, *O*cular hypertelorism, *P*ulmonic stenosis, *A*bnormal genitalia, *R*etardation of growth, and sensorineural *D*eafness [1,2]. This disease was also known as Multiple Lentigines syndrome, Cardio-cutaneous syndrome, Moynahan syndrome, Lentiginosis profusa and Progressive Cardiomyopathic Lentiginosis.

## Historical notes

LS was first reported by Zeisler and Becker in 1936, in a 24-year-old woman presenting with multiple lentigines, increasing in number from birth to puberty, pectus carinatum, hypertelorism and prognathism [3]. A few decades later, Gorlin *et al*. reviewed this disorder and coined the LEOPARD acronym supporting the concept of a more generalised condition [1].

## Epidemiology

LS is a rare condition, but the exact birth prevalence is unknown. Not less than 200 patients have been reported and two reviews published [1,4]. Within the group of the so called 'neuro-cardio-facial-cutaneous' (NCFC) syndromes, LS is probably the second most common disorder after Noonan syndrome (NS) [5]. However, LS is likely underdiagnosed or misdiagnosed as many of its features are mild and the correct diagnosis might be missed in the absence of lentiginosis.

## Clinical description

LS is characterised by the presence of multiple lentigines, although these patients are showing a wide spectrum of features with marked variation in expression [1,2,4,6]. As mnemonically suggested by the acronym, the main features include multiple lentigines, facial dysmorphisms, cardiac anomalies, electrocardiographic (ECG) conduction abnormalities, retardation of growth, abnormal genitalia and sensorineural deafness.

### Facies

**Figure 1:**
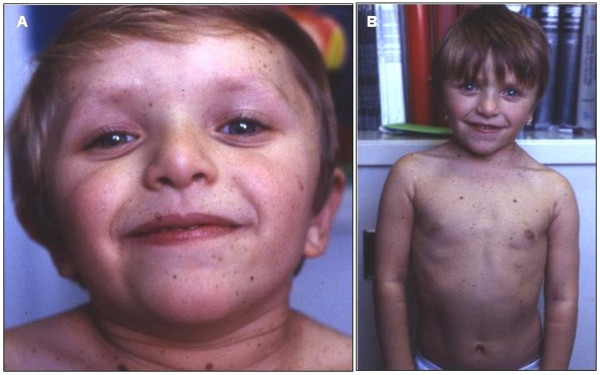
Five year-old girl with multiple lentigines, *cafè-au-lait *spots and dysmorphic features. 1A. Note the hyperthelorism, ptosis, blue eyes and low set dysmorphic ears. 1B. Note the short neck with pterigium colli, teletelia, pectus excavatum and diffuse lentiginosis on the trunk.

Facial dysmorphisms are characteristic and change considerably with age, being less striking at birth and in the first infancy, with the most characteristic features becoming evident during childhood (Figure 1). Dysmorphic features can occur or be only mildly expressed in the newborns and infants [7]. Hypertelorism is virtually present in all cases, and flat nasal bridge and dysmorphic ears in about 87% of the patients. Additional, less frequent, features include palpebral ptosis, thick lips and low-set ears with overfolded helix (50%), large and everted ears, pterygium colli or redundant neck skin (37%) [7]. Adult patients usually manifest hypertelorism, palpebral ptosis, low-set ears, deep nasal-labial folds and premature skin wrinkling.

### Cardiovascular system

Electrocardiographic anomalies and progressive conduction anomalies are the most common heart defects [4,6-8]. A superiorly oriented mean QRS axis in the frontal plane is frequently observed, even in the absence of structural cardiac abnormalities, and does represent a useful diagnostic handle. A cardiological review indicated that ECG abnormalities occur in about 75% of the patients, including left or biventricular hypertrophy in 46% of them, often in association with q waves (19%), prolonged QTc (23%) and repolarisation abnormalities (42%) [8]. Conduction defects are found in 23% of the patients and p wave abnormalities in 19%. On the whole, about 70% of LS individuals display cardiac defects [[8]; personal observation]. Previous reports suggested that pulmonary valve stenosis (PVS), with or without dysplasia, is the most common defect (40%) [4,6]. However, current data points to a consistently lower figure for this defect (10–20%) [8,9]. Conversely, hypertrophic cardiomyopathy (HCM) is the most frequent anomaly and represents the only life-threatening problem in these patients. HCM, which in general is asymmetric and involves the left ventricle, is detected in up to 80% of the subjects with cardiac defect, and may associate with significant left ventricular outflow tract obstruction in up to 40% of the cases [[8-10]; personal data]. Fatal events and sudden death have been reported in LS patients with HCM [7,8,11,12]. HCM can be congenital, but frequently manifests during the second infancy [7,8,12]. The onset of HCM usually precedes multiple lentigines, but the hypertrophic process may start or often worsen in parallel with lentigines appearance. Mitral valve prolapse, clefting or other morphological abnormalities have been found in up to 42% of cases [8]. Less frequent heart defects are atrial and atrioventricular septal defects, coronary artery abnormalities, apical aneurysm and non compaction of the left ventricle, multiple ventricular septal defects, isolated left ventricular enlargement and endocardial fibroelastosis [[6,8]; personal observation].

### Skin

**Figure 2:**
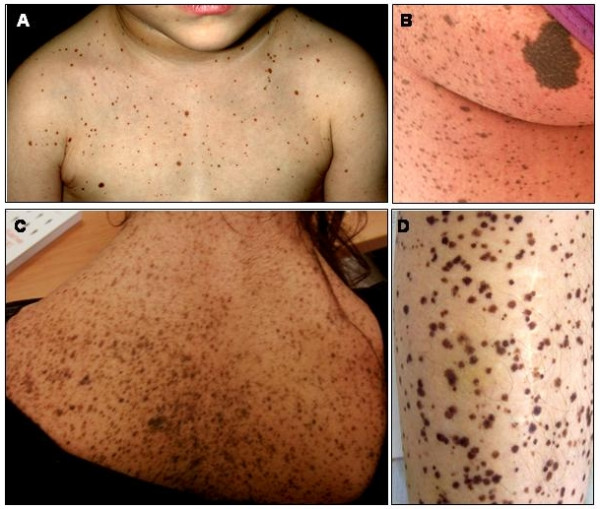
Skin features of LS individual at different ages. 2A: Numerous lentigines in the upper part of the trunk in a 2 year old child with a *PTPN11 *gene mutation. 2B. Lentigines and a large *cafè-au-lait *spot (28 year-old female patient). 2C. A 28 year old female patient with thousands of lentigines scattered all over the neck and back. Note the pterigium colli. 2D. Multiple lentigines on the lower leg (37 year-old male patient).

Multiple lentigines are a distinct feature of LS, even if they may be absent in young patients and, quite exceptionally also in subjects older than 5 years [[10]; personal observation] (Figure 1 and 2). Lentigines are flat, black-brown macules, dispersed mostly on the face, neck, and upper part of the trunk, but sparing the mucosae. In general, lentigines appear at 4–5 years and increase into the thousands until puberty, independently from sun exposure (Figure 2). On histological examination, lentigines are characterised by pigment accumulation in the dermis and deeper epidermal layers, with increased number of melanocytes per unit skin area. The *cafè-au-lait spots *(CLS), occurring in about half of the patients, are similar to those found in Neurofibromatosis type 1 (NF 1), although they appear much darker in the dark skinned individuals (Figure 2). CLS may be congenital or precede the appearance of lentigines [[10]; personal observation]. Occasional hypopigmented skin areas may also be observed [13].

### Weight and length

Birth weight is normal or above the average in one third of the newborns [10]. Subsequently, LS patients show retardation of growth, with 25% of cases below the 3^rd ^centile in height and 85% of adults below the 25th centile [[4,14]; personal observation].

### Skeletal anomalies

Thorax anomalies, including broad chest, pectus carinatum or excavatum are found in up to 75% of the newborns [10]. Mandibular prognathism, winging of the scapulae, scoliosis, joint hyperflexibility and other findings are less common [14].

### Genital and urinary tract anomalies

Bilateral cryptorchidism occurs in about 50% of males, but hypospadia and genital hypoplasia are also frequent. Delayed puberty and hypoplastic ovary have been reported in females. More cases are transmitted through affected mothers suggesting a reduced male fertility. Renal anomalies, including horseshoe kidney, are rare [4,10].

### Hearing loss

Sensorineural deafness occurs in about 15–25% of patients [6,9]. Most cases are diagnosed at birth or during childhood, but deafness may develop also later in life.

### Neurological abnormalities

Hypotonia is common in the newborn and can result in delayed psychomotor development [10]. Mild learning difficulties are reported in about 30% of the cases, while mental retardation is rare [[4,10]; personal observation].

### Tumours

Haematological complications, such as myelodysplasia, acute myelogenous leukaemia and neuroblastoma, have been described in a few patients [4,15,16]. Malignant melanoma was diagnosed in a patient with a germline *PTPN11 *and a somatic *BRAF *mutation [17]. Bilateral choristomas have been reported in a 5-year-old girl [18].

## Aetiology

LS may be sporadic or inherited as an autosomal dominant fully penetrant trait. In approximately 85% of the patients with a definite diagnosis of LS, a missense mutation is found in the *PTPN11 *gene, located on chromosome 12q24.1 [9,19]. The *PTPN11 *gene encodes for the SRC homology 2 (SH2) domain-containing PTPase (SHP2) protein, characterised by two tandemly arranged SH2 (N-SH2 and C-SH2) domains and one protein tyrosine phosphatase (PTP) domain. SHP2 functions as a cytoplasmic signalling transducer downstream of multiple receptors for growth factors, cytokines and hormones, with a particular role through the RAS-mitogen activated protein kinase (MAPK) pathway [20,21]. To the best of our knowledge, 11 different missense *PTPN11 *mutations, in exon 7, 12 and 13 (Tyr279Cys/Ser, Ala461Thr, Gly464Ala, Thr468Met/Pro, Arg498Trp/Leu, Gln506Pro, and Gln510Glu/Gly), have been reported so far, two of which (Tyr279Cys and Thr468Met) occur in about 65% of the cases [[9,10,22-29], personal data]. Germinal mutations in the *PTPN11 *gene are also responsible for about 40–50% of Noonan (NS) and Noonan-like/Multiple Giant Cell lesions syndrome cases [30,31]. Known changes appear to be exclusive for NS or LS, leading to specific genotype-phenotype correlations between these two disorders [23,31]. Among patients with *PTPN11 *mutations, an association between exon 7 and 12 mutations and HCM, and between exon 8 mutations and PVS, has been established [23]. LS patients without *PTPN11 *mutations show a higher prevalence of ECG abnormalities and left ventricle hypertrophy [8]. Analyses of the natural history of HCM in LS patients with different genotypes indicate that patients without *PTPN11 *mutations show a higher frequency of family history of sudden death, increased left atrial dimensions, bradyarrhythmias and other adverse arrhythmic and nonarrhythmic events [12]. Mutations affecting exon 13 in the *PTPN11 *gene are often associated with an important cardiac phenotype, characterised by rapidly progressive severe biventricular obstructive HCM, often with prenatal onset, and with serious cardiac complications during follow-up (heart failure, septal myectomy, and sudden death) [12,28,32]. Analysis of personal cohorts of LS patients indicate that mutation of the Thr468 residue is less frequently associated with short stature, compared to mutation of the Tyr279 residue (26% *vs*. 47%), in which also deafness is more common (24% *vs*. 9%) [personal data]. This data confirm a previous observation of less adverse effects of the Thr468Met mutation on body growth and cardiac development, with lower prevalence of PVS in these patients [33].

Although LS and NS are clinically overlapping conditions, nosological splitting is well supported by distinct functional effects of the disease causing mutation: gain of function in NS and reduced protein tyrosine phosphatase activity in LS, suggestive of a dominant-negative effect [34,35].

Genetic heterogeneity was supported by linkage analysis [36], and recently confirmed by the identification of *RAF1 *gene mutations in two out of six *PTPN11 *mutation negative LS patients [37]. RAF1 protein is one of the three mammalian RAF isoforms (ARAF, BRAF and CRAF or RAF1), threonine-serine protein kinases with nonredundant developmental functions, acting downstream of RAS [38]. *RAF1 *gene mutations are also responsible for a subset of NS, 75% of which develop HCM [37,39]. The two LS subjects carrying the Leu613Val and Ser257Leu changes disclosed a full blown LS phenotype, with multiple lentigines, CLS, HCM and delayed puberty [37]. Pandit *et al*. investigated the functional effect of different RAF1 mutants, including the Leu613Val change, and showed that those associated with HCM had increased kinase activity and enhanced ERK activation [37]. These data reinforce the role of increased RAS signalling in cardiomyocyte hypertrophy pathogenesis and suggest that LS pathogenesis should not be simply related to a reduced RAS signal transduction [37,40].

About 5% of LS patients of the reported series do not have *PTPN11 *or *RAF1 *mutations. Analysis of additional genes encoding for member or the RAS pathway will likely expand the LS genetic heterogeneity.

Distinct missense *PTPN11 *gene mutations occur as somatic events in myeloid or lymphoid malignancies [[34]; Cosmic database]. Both the spectrum and the distribution of these *PTPN11 *mutations are different from those documented in LS and related disorders.

## Diagnosis and differential diagnosis

LS is a rare autosomal dominant disease, with high penetrance and marked variable expression, mainly characterised by short stature, facial dysmorphisms, cardiac anomalies and hyperpigmented skin lesions, specifically multiple lentigines and CLS [1,2,6]. According to Voron *et al*., clinical diagnosis of LS may be suspected in the presence of multiple lentigines and two cardinal features [4]. In the absence of lentiginosis, three features in the patient and the presence of an affected close relative are diagnostic. Since some features manifest with advancing age, the diagnosis may be problematic in very young patients with only partial phenotypes. However, molecular testing is supportive in this difficult task [9]. Digilio *et al*. suggested that diagnosis of LS in the first months of age can be clinically suspected in the presence of three main features, including HCM, distinct facial dysmorphisms and CLS [10].

LS is one of the so called 'neuro-cardio-facial-cutaneous'(NCFC) syndromes [5], which include some overlapping disorders, such as NS, Neurofibromatosis type 1, Costello syndrome, Cardiofaciocutaneous syndrome and LS itself, all caused by mutations in some components of the Ras signalling pathway. These subjects display facial anomalies, heart defects and growth retardation, often associated with skin, skeletal and genital anomalies, and variable degree of mental retardation. In addition, each of these conditions present a few distinct features that are useful handles for differential diagnosis.

Except for its most striking feature, *i.e*. the multiple lentigines, LS largely overlaps with NS [41,42]. While NS patients show more conspicuous facial features in infancy and childhood, PVS (single-ventricle physiology) is the most frequent cardiac defect in this condition, and skin anomalies and deafness have been rarely reported. Conversely, diagnostic clues of LS are the cutaneous manifestations, such as CLS and multiple lentigines, HCM and deafness. The phenotypic overlap between NS and LS may complicate the differential diagnosis in young individuals who have not yet developed lentigines. Molecular diagnosis and long term follow-up are critical in these individuals. In fact, re-examination later in life may reveal the presence of CLS or lentigines, as for a patient with the Tyr279Cys mutation reported by Tartaglia *et al*. [31,43]. Likewise, detection of a LS-related mutation should shift the diagnosis toward this condition, as suggested by Digilio *et al*. for the patient reported by Takahashi and colleagues [8,28,32].

LS also displays an important phenotypic overlap with Neurofibromatosis-Noonan syndrome (NFNS), a clinical entity manifesting with the association of facial and cardiac characteristics of NS with clinical features of neurofibromatosis 1, including CLS, neurofibromas, central nervous system and skeletal anomalies [44,45]. NFNS is largely caused by *NF1 *gene mutations [46]. Nevertheless, NFNS may have different potential aetiologies and NFNS phenotype has been described also in patients with mutations in both the *NF1 *and *PTPN11 *genes [47] as well as in neurofibromatosis 1 (NF1) patients with Noonan-like features [48,49]. In addition, patients with NS-related gene mutations can have pigmentary skin manifestations similar to those present in neurofibromatosis 1. Mutation-based differential diagnosis in patients with borderline clinical manifestations is warranted [50].

## Genetic counselling

LS is caused by heterozygous missense mutations in autosomal genes. Familial cases are commonly reported, and prevalence of transmitting mothers might be related to reduced male fertility. Genetic counselling should include:

• revision of a three generation family tree, with specific enquiries for skin and cardiac anomalies, short stature and learning difficulties;

• revision of pregnancy and developmental history and schooling;

• examination of growth parameters, facial dysmorphisms, skin, skeleton, joints, heart and external genitalia;

• complete clinical and cardiological examination of parents, inclusive of echocardiogram and ECG, if possible;

• revision of natural history of the condition, its manifestation and clinical variability, occurrence and recurrence risks, and the eventual recommendation for clinical and molecular investigations to confirm the diagnosis;

• management and follow-up, inclusive of available treatments and interventions.

If one of the parents is affected, a 50% recurrence risk is appropriate. Germinal mutations and autosomal recessive inheritance have not been reported so far. Accordingly, in case of identified *de novo *mutation in sporadic patients, the recurrence risk for siblings is marginal. Molecular investigation should take into account the *PTPN11 *gene screening first, and then the *RAF1 *gene screening in *PTPN11*-negative individuals.

## Antenatal diagnosis

The prenatal differential diagnosis between NS and LS may be extremely difficult. LS should be suspected in foetuses with a normal karyotype and HCM. If LS is suspected, physical examination of the parents is indicated. If one parent is affected, obstetric ultrasound at different timings and foetal echocardiography at 20 weeks' of gestations is indicated. A DNA test for mutation analysis can be carried out on chorionic villi or amniotic fluid samples.

## Management, treatment and prognosis

Clinical management, follow-up and treatment of LS patients greatly overlaps that of NS [41,42]. However, a few issues need to be addressed differently, taking into account the specific clinical problems and needs of LS individuals. Baseline studies at diagnosis should include a complete clinical examination, cardiological, genitourinary and neurological evaluations and hearing assessment. Laboratory studies should include molecular analysis of the *PTPN11 *and *RAF1 *genes.

In general, long-term prognosis of LS patients is favourable. In case of cardiac anomalies at diagnosis, a periodic assessment should be performed as recommended by the cardiologist. Otherwise, a complete cardiological assessment should be performed annually and particularly at the appearance of multiple lentigines. Mild PVS has a good prognosis, while severe valvular dysplasia of the pulmonary valve may recommend valvulotomy or valvulectomy [8]. As suggested by Limongelli *et al*., pathologic and clinical findings may be similar in familial HCM and left ventricular hypertrophy associated to LS [8]. Accordingly, LS patients with ventricular hypertrophy may follow familial HCM algorithms [51]. A beta-blockade or calcium channel blockers therapy could be indicated in the case of a significant gradient between the left ventricle and the aorta. In the absence of any improvement, surgical removal of the left ventricular outflow obstruction is indicated. Fatal events occurring in patients with HCM recommend careful risk assessment and prophylaxis against sudden death in patients at risk [8,12,13]. Isolated conduction defects should be monitored annually and treated as in the general population. Annual hearing assessment should be performed until adulthood and hearing aids indicated, if needed. During follow-up, growth parameters should be monitored as in NS [41,42]. If growth hormone (GH) therapy is started, the cardiac status should be ongoing assessed, in particular in the presence of HCM. In the case of multiple lentigines or CLS, total UVA-UVB protection should be indicated. Genitourinary, musculoskeletal, neurological and orthodontic anomalies should be monitored and treated as for NS [41,42]. In the presence of developmental delay or difficulties at school, an infant stimulation program and alternate teaching method should be initiated. Hypotonia usually is benefiting of physical and occupational therapies. On the whole, most adults with LS do not require special medical care. Males with cryptorchidism in childhood may have fertility problems in adulthood.

## Unresolved questions

The LS phenotype is extremely heterogeneous, ranging from adults with mild facial features and multiple lentigines, to patients with severe HCM, mental retardation, deafness and additional defects. Genotype-phenotype correlation analysis and functional studies are providing answers to these questions. In early childhood and before the appearance of lentigines, diagnosis of LS is sometimes difficult because of the overlap with NS and NFNS. In these patients, a mutation-based diagnosis is recommended.

Presence of patients without *PTPN11 *and *RAF1 *mutations further expand genetic heterogeneity of LS and points towards other genes likely involved in the same RAS-MAPK pathway.

## Abbreviations

LS: LEOPARD syndrome; CLS: c*afè-au-lait *spots; HCM: hypertrophic cardiomyopathy; NCFC: neuro-cardio-facial-cutaneous; PVS: pulmonary valve stenosis; NS: Noonan syndrome; NFNS: Neurofibromatosis- Noonan syndrome;

## Competing interests

The authors declare that they have no competing interests.

## Authors' contributions

AS participated in the design of the study, carried out the molecular genetic studies, analyzed clinical and molecular data, and drafted the manuscript, MCD carried out analysis of clinical data and helped to draft the manuscript, BD conceived the study, participated in its design and coordination, and helped to draft the manuscript. All authors read and approved the final manuscript.

## Consent

Written consent was obtained from the patients or by the patient's relative for pubblication. A copy of the written consent is available for review by the Authors of this manuscript.
